# Supplementary material for: Changes in the Density and Composition of Rhizosphere Pathogenic Fusarium and Beneficial Trichoderma Contributing to Reduced Root Rot of Intercropped Soybean
Source: Pathogens. 2022 Apr 16;11(4):478. doi: 10.3390/pathogens11040478 (PMC9031213; doi:10.3390/pathogens11040478)
Supplement: Supplementary file 1 [file pathogens-11-00478-s001.zip › pathogens-1638394-supplementary.pdf]

Supplementary Materials Tables and Figures

Table S1. Primers for PCR amplification of *Fusarium* spp. and *Trichoderma* spp.

Fungi	Primer	Primer sequence	Denature temperature	Target fragments
Fusarium spp.	EF1	ATGGGTAAGGARGACAAGAC	55	*EF-1α*
	EF2	GGARGTACCAGTSATCATGTT		
	RPB2-5f2<br>RPB2-7cr	GGGGWGAYCAGAAGAAGGC<br>CCCATRGCTTGYTTRCCCAT	55	*RPB2*
Trichoderma spp.	*ITS*1<br>*ITS*4	TCCGTAGGTGAACCTGCGG<br>TCCTCCGCTTATTGATATGC	58℃	*ITS*
	EF-728F<br>EF1LLErev	CATCGAGAAGTTCGAGAAGG<br>AACTTGCAGGCAATGTGG	55℃	*EF-1α*
	fRPB2-5F<br>fRPB2-7cR	GAYGAYMGWGATCAYTTYGG<br>CCCATRGCTTGYTTRCCCAT	55℃	*RPB2*

Notes: merge bases, Y=(C/T), M=(A/C), W=(A/T), R=(A/G); *ITS*: 5.8S ribosomal RNA gene; *EF-1α*: translation elongation factor alpha; *RPB2:* RNA polymerase II subunit gene.

Table S2. Information of referred isolates used for phylogenetic analysis of *Fusarium* and *Trichoderm* species.

Fusarium and *Trichoderma* isolates	*Fusarium* and *Trichoderma* species	Gene bank Accession numbers		
		RPB2	***EF- 1α***	***ITS***
NRRL 34034 *F. sp. FIESC*	*Fusarium incarnatum-equiseti* species complex, FIESC	GQ505814	GQ505636	-
NRRL 43637 *FIESC*	*FIESC*	GQ505842	GQ505664	-
NRRL 28058 *F. commune*	*Fusarium commune*	MH582180	MH582349	-
NRRL 32709 *F. sp FSSC*	*Fusarium solani* species complex*, FSSC*	FJ240397	DQ247029	-
NRRL 32829	*FSSC*	FJ240401	DQ247136	-
NRRL 32812	*FSSC*	FJ240409	DQ247120	-
NRRL 32762	*FSSC*	FJ240407	DQ247079	-
NRRL 25388	*FSSC*	MH582411	MH582421	-
NRRL 22858	*FSSC*	MH582230	MH582424	-
NRRL 22856 *F. solani*	*FSSC*	MH582228	MH582423	-
NRRL 22855	*FSSC*	MH582227	MH582422	-
NRRL 28056	*Fusarium oxysporum*	MH582140	MH582353	-
NRRL 28057	*F.oxysporum*	MH582354	MH582140	-
NRRL 28055	*F.oxysporum*	MH582152	MH582351	-
NRRL 38288	*F.oxysporum*	MH582352	MH582126	-
NRRL 52754	*Nectriaceae spp.*	JF741155	JF740829	-
GJS04-186	*Trichoderma afroharzianum*	FJ442691	FJ463301	FJ442265
GJS04-197	*T. afroharzianum*	FJ442740	FJ463302	FJ442214
NAIMCC-F-01938	*T. afroharzianum*	KY419895	KY419891	KY419889
GJS 01287	*Trichoderma virens*	EU341804	AY750894	DQ083023
TRS106	*T.virens*	KP009093	KP008854	KP009291
TRS112	*T.virens*	KP009098	KP008860	KP009296
HZA14	*T.virens*	MH647804	MH647804	MH624149
G.J.S 00-18	*Trichoderma harzianum*	FJ442750	AF443946	AF443929
DIS 218E	*T.harzianum*	FJ442793	FJ463310	FJ442220
T18	*T.harzianum*	KX632549	KX632606	KX632492
DAOM166989	*Trichoderma longib*	EU338339	EU33833	EU330961

Notes: *ITS:* 5.8S ribosomal RNA gene; *EF-1α*: translation elongation factor alpha; *RPB2*: RNA polymerase II subunit gene.

Table S3. Information of *Fusarium* isolates and GenBank accession information.

Cropping pattern	Soil type	Isolates code	GenBank accession no.		Identified *Fusarium* species or species complex
			EF-1α	*RPB2*	
Intercropping	IDR	RA1-8	MW319811	MW364892	FIESC
		RA1-9	MW319765	MW368344	*F. oxysporum*
		RA1-10	MW319766	MW368345	*F. oxysporum*
		RA1-17	MW319767	MW368380	*F. oxysporum*
		RA1-57	MW345822	MW368346	*F. oxysporum*
		RA1-1	MW331879	MW375137	FSSC
		RA1-2	MW331880	MW375343	FSSC
		RA1-3	MW331881	MW375138	FSSC
		RA1-4	W331882	MW375139	FSSC
		RA1-5	MW331883	MW375140	FSSC
		RA1-6	MW331884	MW375141	FSSC
		RA1-11	MW331885	MW375142	FSSC
		RA1-20	MW331886	MW375143	FSSC
		RA1-21	MW331887	MW375144	FSSC
		RA1-22	MW331888	MW375145	FSSC
		RA1-26	MW331889	MW375326	FSSC
		RA1-27	MW331890	MW375146	FSSC
		RA1-33	MW331891	MW375147	FSSC
		RA1-41	MW331892	MW375148	FSSC
		RA1-42	MW331893	MW375149	FSSC
		RA1-47	MW331894	MW375150	FSSC
		RA1-49	MW331895	MW375151	FSSC
		RA1-52	MW331896	MW375152	FSSC
		RA1-55	MW331897	MW375153	FSSC
		RA1-56	MW331898	MW375154	FSSC
		A1-1	MW331965	MW375210	FSSC
		A1-2	MW331966	MW375211	FSSC
		A1-4	MW331967	MW375323	FSSC
		A1-6	MW331968	MW375212	FSSC
		A1-7	MW331969	MW375213	FSSC
		A1-9	MW331970	MW375214	FSSC
		A1-10	MW331971	MW375215	FSSC
		A1-12	MW331972	MW375216	FSSC
		A1-23	MW331973	MW375217	FSSC

		A1-24	MW331974	MW375218	FSSC
		A1-25	MW331975	MW375219	FSSC
		A1-35(1)	MW331976	MW375341	FSSC
		A1-44	MW331977	MW375221	FSSC
		A1-47	MW331978	MW375325	FSSC
		A1-48	MW331979	MW375222	FSSC
		A1-52	MW331980	MW375223	FSSC
		A1-55	MW331981	MW375224	FSSC
		A1-56	MW331982	MW375225	FSSC
		A1-57	MW331983	MW375226	FSSC
		A1-59	MW331984	MW375227	FSSC
	IHR	RA2-45	MW319812	MW364893	FIESC
		RA2-11	MW319768	MW368382	*F. oxysporum*
		RA2-13	MW319769	MW368347	*F. oxysporum*
		RA2-25	MW319770	MW368348	*F. oxysporum*
		RA2-50	MW319771	MW368381	*F. oxysporum*
		A2-31	MW345823	MW368349	*F. oxysporum*
		A2-58	MW319797	MW368350	*F. oxysporum*
		RA2-1	MW331901	MW375156	FSSC
		RA2-2	MW331902	MW375157	FSSC
		RA2-3	MW331903	MW375158	FSSC
		RA2-4	MW331904	MW375327	FSSC
		RA2-18	MW331906	MW375336	FSSC
		RA2-19	MW331907	MW375159	FSSC
		RA2-21	MW331908	MW375160	FSSC
		RA2-22	MW331909	MW375337	FSSC
		RA2-27	MW331910	MW375161	FSSC
		RA2-31	MW331911	MW375162	FSSC
		RA2-44	MW331912	MW375163	FSSC
		RA2-46	MW331913	MW375164	FSSC
		RA2-47	MW331914	MW375165	FSSC
		RA2-49	MW331915	MW375166	FSSC
		RA2-52	MW331916	MW375167	FSSC
		RA2-53	MW331917	MW375168	FSSC
		A2-1	MW362315	MW375229	FSSC
		A2-2	MW362316	MW375230	FSSC
		A2-3	MW362317	MW375231	FSSC
		A2-21	MW362318	MW375232	FSSC
		A2-23	MW331985	MW375233	FSSC
		A2-40	MW362319	MW375234	FSSC
		A2-54	MW331986	MW375228	FSSC
Monocultur		RB1-25	MW314606	MW325768	*F. commune*

e	MDR	RB1-7	MW319777	MW368376	*F. oxysporum*
		RB1-8	MW319778	MW368377	*F. oxysporum*
		RB1-10	MW319779	MW368355	*F. oxysporum*
		RB1-15	MW319780	MW368378	*F. oxysporum*
		RB1-26	MW319781	MW368383	*F. oxysporum*
		RB1-29	MW319782	MW368379	*F. oxysporum*
		RB1-37	MW319783	MW368356	*F. oxysporum*
		RB1-42	MW319784	MW368357	*F. oxysporum*
		RB1-48	MW319785	MW368358	*F. oxysporum*
		RB1-53	MW319786	MW368359	*F. oxysporum*
		RB1-60	MW319787	MW368360	*F. oxysporum*
		B1-12	MW319799	MW368361	*F. oxysporum*
		B1-28	MW319800	MW368362	*F. oxysporum*
		B1-49	MW319802	MW368363	*F. oxysporum*
		RB1-4	MW331923	MW375172	FSSC
		RB1-5	MW331924	MW375173	FSSC
		RB1-17	MW331925	MW375174	FSSC
		RB1-18	MW331926	MW375175	FSSC
		RB1-28	MW331927	MW375176	FSSC
		RB1-40	MW331928	MW375177	FSSC
		RB1-43	MW331929	MW375178	FSSC
		RB1-44	MW331930	MW375179	FSSC
		RB1-55	MW331931	MW375180	FSSC
		RB1-56	MW331932	MW375330	FSSC
		B1-1	MW331989	MW375251	FSSC
		B1-3	MW362311	MW375253	FSSC
		B1-8	MW332026	MW375255	FSSC
		B1-10	MW332027	MW375328	FSSC
		B1-14	MW332007	MW375256	FSSC
		B1-21	MW362312	MW375257	FSSC
		B1-22	MW332028	MW375258	FSSC
		B1-24	MW332008	MW375259	FSSC
		B1-25	MW331990	MW375260	FSSC
		B1-32	MW362310	MW375261	FSSC
		B1-35	MW332030	MW375262	FSSC
		B1-36	MW332009	MW375263	FSSC
		B1-38	MW331991	MW375264	FSSC
		B1-39	MW331992	MW375344	FSSC
		B1-41	MW362313	MW375266	FSSC
		B1-43	MW331993	MW375268	FSSC
		B1-45	MW332010	MW375269	FSSC
		B1-47	MW332031	MW375329	FSSC

	MHR	RB2-13	MW314608	MW325770	*F. commune*
		RB2-16	MW319788	MW368364	*F. oxysporum*
		RB2-17	MW319789	MW368365	*F. oxysporum*
		RB2-37	MW319790	MW368366	*F. oxysporum*
		RB2-49	MW319791	MW368367	*F. oxysporum*
		RB2-60	MW319793	MW368368	*F. oxysporum*
		RB2-1	MW331933	MW375181	FSSC
		RB2-2	MW331934	MW375182	FSSC
		RB2-5	MW331935	MW375338	FSSC
		RB2-8	MW331936	MW375183	FSSC
		RB2-12	MW331937	MW375184	FSSC
		RB2-19	MW331938	MW375185	FSSC
		RB2-21	MW331939	MW375335	FSSC
		RB2-23	MW331940	MW375186	FSSC
		RB2-24	MW331941	MW375187	FSSC
		RB2-25	MW331942	MW375188	FSSC
		RB2-26	MW331943	MW375346	FSSC
		RB2-27	MW331944	MW375189	FSSC
		RB2-28	MW331945	MW375190	FSSC
		RB2-40	MW331946	MW375191	FSSC
		RB2-41	MW331947	MW375192	FSSC
		RB2-42	MW331948	MW375339	FSSC
		RB2-43	MW331949	MW375193	FSSC
		RB2-44	MW331950	MW375194	FSSC
		RB2-46	MW331951	MW375195	FSSC
		RB2-47	MW331952	MW375340	FSSC
		RB2-52	MW331953	MW375196	FSSC
		RB2-53	MW331954	MW375197	FSSC
		RB2-55	MW331955	MW375198	FSSC
		B2-5	MW331994	MW375331	FSSC
		B2-14	MW332032	MW375332	FSSC
		B2-29	MW332011	MW375279	FSSC
		B2-34	MW332033	MW375284	FSSC
		B2-36	MW331996	MW375285	FSSC
		B2-37(1)	MW332034	MW375286	FSSC
		B2-38(2)	MW331997	MW375287	FSSC
		B2-39	MW331998	MW375288	FSSC
		B2-44	MW331999	MW375289	FSSC
		B2-48	MW332036	MW375290	FSSC
		B2-51(1)	MW332000	MW375291	FSSC
		B2-52	MW332038	MW375333	FSSC
		B2-53	MW332001	MW375292	FSSC

		B2-54	MW332002	MW375293	FSSC
		B2-55(2)	MW332039	MW375294	FSSC
		B2-56	MW332041	MW375296	FSSC
		B2-58	MW362314	MW375297	FSSC
		B2-60	MW332042	MW375334	FSSC

Notes: *EF-1α*: translation elongation factor alpha; *RPB2:* RNA polymerase II subunit gene; IDR is the abbreviation of maize-soybean intercropping rhizosphere disease soil, IHR stands for maize-soybean intercropping rhizosphere healthy soil, MDR stands for the disease rhizosphere soil of soybean monoculture, while MHR stands for the healthy rhizosphere soil of soybean monoculture.

Table S4. Information of *Trichoderma* isolates from different soil samples in monoculture and intercropping.

Cropping pattern	Soil type	Isolate code	GenBank accession no.			Identified *Trichoderma* species
			ITS	EF-*1α*	*RPB2*	
Intercropping	IDR	TRA1-9	MW325931	MW325735	-	*T. virens*
		RA1-20	MW325943	MW345821	-	*T. virens*
		TRA1-35	MW325940	MW325741	MW325766	*T. virens*
		TA1-36	MW325942	MW325743	-	*T. virens*
		TRA1-50	MW325941	MW325742	MW325767	*T. virens*
		TA1-11	MW338651	-	-	*T. virens*
		TRA1-28	MW325772	MW267259	MW325744	*T. afroharzianum*
		TRA1-31	MW325778	MW267265	MW325745	*T. afroharzianum*
		TRA1-32	MW325773	MW267260	MW325747	*T. afroharzianum*
		TA1-49	MW325779	MW267266	MW325749	*T. afroharzianum*
		TRA1-1	MW325784	MW267302	MW331755	*T. harzianum*
		TRA1-2	MW325832	MW267346	MW331801	*T. harzianum*
		TRA1-3	MW325785	MW267303	MW331756	*T. harzianum*
		TRA1-4	MW325786	MW267304	MW331757	*T. harzianum*
		TRA1-5	MW325787	MW267305	MW331758	*T. harzianum*
		TRA1-6	MW325788	MW267306	MW331759	*T. harzianum*
		TRA1-7	MW325789	MW267307	MW331760	*T. harzianum*
		TRA1-8	MW325790	MW267308	MW331761	*T. harzianum*
		TRA1-12	MW325833	MW267347	MW331802	*T. harzianum*
		TRA1-13	MW325791	MW267309	MW331762	*T. harzianum*
		TRA1-15	MW325834	MW267348	MW331803	*T. harzianum*
		TRA1-16	MW325792	MW267310	MW331763	*T. harzianum*
		TRA1-17	MW325835	MW267349	MW331804	*T. harzianum*
		TRA1-19	MW325836	MW267350	MW331805	*T. harzianum*
		TRA1-21	MW325793	MW267311	MW331764	*T. harzianum*
		TRA1-25	MW325794	MW267312	MW331765	*T. harzianum*

		TRA1-26	MW325837	MW267351	MW331806	*T. harzianum*
		TRA1-27	MW325838	MW267352	MW331807	*T. harzianum*
		TRA1-30	MW325839	MW267353	MW331808	*T. harzianum*
		TRA1-33	MW325840	MW267354	MW331809	*T. harzianum*
		TRA1-34	MW325946	MW325729	MW325750	*T. harzianum*
		TRA1-39	MW325795	MW267313	MW331766	*T. harzianum*
		TRA1-41	MW325841	MW267355	MW331810	*T. harzianum*
		TRA1-42	MW325947	MW325730	MW325751	*T. harzianum*
		TRA1-44	MW325842	MW267356	MW331811	*T. harzianum*
		TRA1-45	MW325843	MW267357	MW331812	*T. harzianum*
		TRA1-46	MW325844	MW267358	MW331813	*T. harzianum*
		TRA1-49	MW325845	MW267359	MW331814	*T. harzianum*
		TA1-6	MW325880	MW267390	MW331849	*T. harzianum*
		TA1-22	MW325881	MW267391	MW331850	*T. harzianum*
		TA1-24	MW325882	MW267392	MW331851	*T. harzianum*
		TA1-46	MW325883	MW267393	MW331852	*T. harzianum*
	IHR	TA2-2	MW325928	MW325733	MW325755	*T. virens*
		TRA2-22	MW325945	-	-	*T. virens*
		TA2-60	MW325944	-	-	*T. virens*
		TRA2-2	MW325846	MW267360	MW331815	*T. harzianum*
		TRA2-3	MW325796	MW267314	MW331767	*T. harzianum*
		TRA2-4	MW325797	MW267315	MW331768	*T. harzianum*
		TRA2-5	MW325798	MW267316	MW331878	*T. harzianum*
		TRA2-6	MW325847	MW267361	MW331816	*T. harzianum*
		TRA2-8	MW325799	MW267317	MW331769	*T. harzianum*
		TRA2-9	MW325848	MW267362	MW331817	*T. harzianum*
		TRA2-10	MW325849	MW267363	MW331818	*T. harzianum*
		TRA2-14	MW325850	MW267364	MW331819	*T. harzianum*
		TRA2-15	MW325851	MW267365	MW331820	*T. harzianum*
		TRA2-16	MW325800	MW267318	MW331770	*T. harzianum*
		TRA2-20	MW325801	MW267319	-	*T. harzianum*
		TRA2-21	MW325852	MW267366	MW331821	*T. harzianum*
		TRA2-23	MW325853	MW267367	MW331822	*T. harzianum*
		TRA2-24	MW325854	MW267368	MW331823	*T. harzianum*
		TRA2-25	MW325855	-	MW331824	*T. harzianum*
		TRA2-26	MW325802	MW267320	MW331771	*T. harzianum*
		TRA2-27	MW325856	MW267369	MW331825	*T. harzianum*
		TRA2-28	MW325803	MW267321	MW331772	*T. harzianum*
		TRA2-29	MW325857	MW267370	MW331826	*T. harzianum*
		TRA2-30	MW325858	MW267371	-	*T. harzianum*
		TRA2-31	MW325859	MW267372	MW331827	*T. harzianum*
		TRA2-32	MW325860	MW267373	MW331828	*T. harzianum*
		TRA2-33	MW325861	MW267374	MW331829	*T. harzianum*

		TRA2-34	MW325862	MW267375	MW331830	*T. harzianum*
		TRA2-36	MW325863	MW267376	MW331831	*T. harzianum*
		TRA2-37	MW325864	MW267377	MW331832	*T. harzianum*
		TRA2-38	MW325804	MW267322	MW331773	*T. harzianum*
		TRA2-40	MW325805	MW267323	MW331774	*T. harzianum*
		TRA2-41	MW325865	MW267378	MW331833	*T. harzianum*
		TRA2-42	MW325866	MW267379	MW331834	*T. harzianum*
		TRA2-46	MW325867	MW267380	MW331835	*T. harzianum*
		TRA2-48	MW325806	MW267324	MW331775	*T. harzianum*
		TRA2-49	MW325868	MW267381	MW331836	*T. harzianum*
		TRA2-50	MW325869	MW267382	MW331837	*T. harzianum*
		TA2-1	MW325884	MW267394	MW331853	*T. harzianum*
		TA2-3	MW325885	MW267395	MW331854	*T. harzianum*
		TA2-9	MW325886	MW267396	MW331855	*T. harzianum*
		TA2-10	MW325887	MW267397	MW331856	*T. harzianum*
		TA2-12	MW325816	MW267331	MW331785	*T. harzianum*
		TA2-13	MW325817	MW267332	MW331786	*T. harzianum*
		TA2-14	MW325888	MW267398	MW331857	*T. harzianum*
		TA2-15	MW325889	MW267399	MW331858	*T. harzianum*
		TA2-17	MW325818	MW267333	MW331787	*T. harzianum*
		TA2-20	MW325890	MW267400	MW331859	*T. harzianum*
		TA2-22	MW325819	-	MW331788	*T. harzianum*
		TA2-23	MW325891	MW267401	MW331860	*T. harzianum*
		TA2-24	MW325892	MW267402	MW331861	*T. harzianum*
		TA2-25	MW325820	MW267334	MW331789	*T. harzianum*
		TA2-27	MW325821	MW267335	MW331790	*T. harzianum*
		TA2-28	MW325893	MW267403	MW331862	*T. harzianum*
		TA2-29	MW325894	MW267404	MW331863	*T. harzianum*
		TA2-30	MW325822	MW267336	MW331791	*T. harzianum*
		TA2-33	MW325895	MW267405	MW331864	*T. harzianum*
		TA2-34	MW325896	MW267406	-	*T. harzianum*
		TA2-36	MW325897	MW267407	MW331865	*T. harzianum*
		TA2-38	MW325898	MW267408	MW331866	*T. harzianum*
		TA2-41	MW325899	MW267409	MW331867	*T. harzianum*
		TA2-42	MW325823	MW267337	MW331792	*T. harzianum*
		TA2-43	MW325900	MW267410	MW331868	*T. harzianum*
		TA2-44	MW325824	MW267338	MW331793	*T. harzianum*
		TA2-46	MW325901	MW267411	MW331869	*T. harzianum*
		TA2-47	MW325902	MW267412	MW331870	*T. harzianum*
		TA2-49	MW325903	MW267413	MW331871	*T. harzianum*
		TA2-50	MW325825	MW267339	MW331794	*T. harzianum*
		TA2-52	MW325904	MW267414	MW331872	*T. harzianum*

		TA2-53	MW325826	MW267340	MW331795	*T. harzianum*
		TA2-54	MW325905	MW267415	MW331873	*T. harzianum*
		TA2-55	MW325827	MW267341	MW331796	*T. harzianum*
		TA2-58	MW325906	MW267416	MW331874	*T. harzianum*
		TA2-59	MW325907	MW267417	MW331875	*T. harzianum*
Monoculture	MDR	TRB1-7	MW325932	MW325737	MW325758	*T. virens*
		TRB1-12	MW325933	MW325738	MW325759	*T. virens*
		TRB1-15	MW325934	MW325739	MW325760	*T. virens*
		TB1-10	MW325780	MW267267	-	*T. afroharzianum*
		TB1-23	MW325781	MW267268	-	*T. afroharzianum*
		TB1-26	MW325782	MW267269	-	*T. afroharzianum*
		TRB1-3	MW325783	MW267330	MW331845	*T. harzianum*
		TB1-1	MW325921	MW267431	-	*T. harzianum*
		TB1-56	MW325922	MW267432	-	*T. harzianum*
	MHR	TRB2-17	MW325926	MW325732	MW325753	*T. virens*
		TB2-7	MW325929	MW325734	MW325756	*T. virens*
		TB2-14	MW325774	MW267261	MW325748	*T. afrohazianum*
		TRB2-2	MW325877	MW267387	MW331846	*T. harzianum*
		TRB2-3	MW325878	MW267388	MW331847	*T. harzianum*
		TRB2-28	MW325879	MW267389	MW331848	*T. harzianum*
		TB2-1	MW325923	MW267433	MW331876	*T. harzianum*
		TB2-2	MW325831	MW267345	MW331800	*T. harzianum*
		TB2-13	MW325924	MW267434	MW331877	*T. harzianum*

Notes: *ITS*: 5.8 S ribosomal RNA gene; *EF-1α*: translation elongation factor alpha; *RPB2*: RNA polymerase II subunit gene; IDR is the abbreviation of maize-soybean intercropping rhizosphere disease soil, IHR stands for maize-soybean intercropping rhizosphere healthy soil, MDR stands for the disease rhizosphere soil of soybean monoculture, while MHR stands for the healthy rhizosphere soil of soybean monoculture.

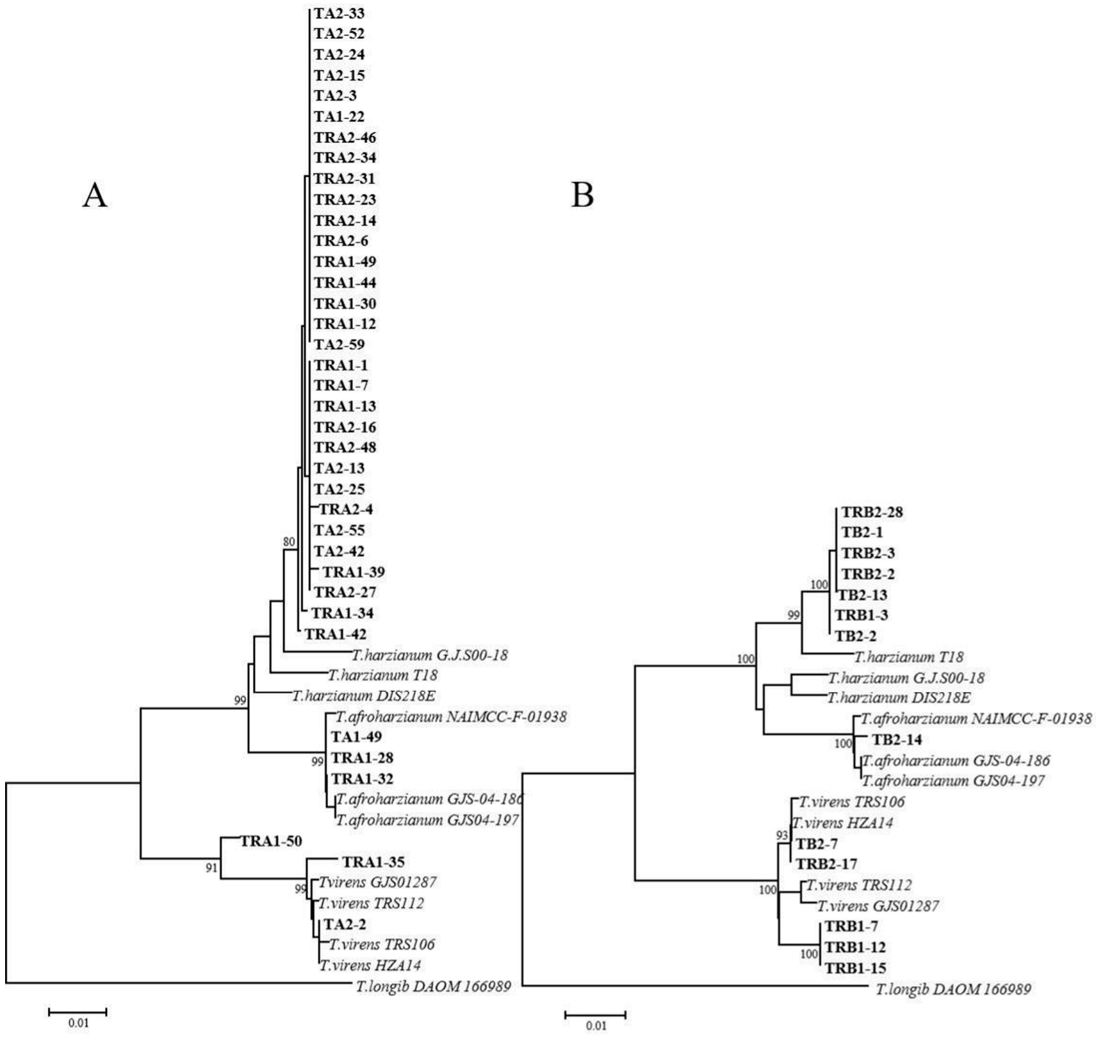

Figure S1. Phylogenetic tree of *ITS* and *EF-1α* of *Trichoderma* spp. isolated from intercropping and monoculture. The Maximum Parsimony based on *ITS* and *EF-1α* genes was constructed for intecropping (A) and monoculture (B), respectively. Bootstrap support values was more than 80% in both trees and obtained from 1000 replications. The *Trichoderma longib* (DAOM 166989) was used as the outgroup isolate.
